# A qualitative investigation into the relationship between stress and eating disordered behaviours in patients with anorexia nervosa

**DOI:** 10.1186/2050-2974-2-S1-O49

**Published:** 2014-11-24

**Authors:** Amanda Hale, Elizabeth Rieger, Janice Russell

**Affiliations:** 1 Australian National University, Canberra, Australia; 2 The University of Sydney, Sydney, Australia

## 

Higher levels of perceived stress and decreased coping skills have been consistently linked to the development, maintenance and relapse of eating disorders. The present qualitative study aims to provide a preliminary investigation into mechanisms linking stress and disordered eating (defined as severe food restriction, bingeing, purging and other compensatory behaviours aimed at weight control) in patients with anorexia nervosa. Semi-structured interviews were conducted with five female patients with anorexia hospitalized in a private inpatient setting. Interpretative phenomological analysis was then used to explore in depth the meaning participants give to their experience of the world. Internalised high expectations and high standards for themselves, interpersonal sensitivities and deficient coping skills emerged as significant sources of stress. Stress itself was found to both act as a cause and a consequence of disordered eating. Subjectively, the participants identified multiple mechanisms linking stress and disordered eating, conceptualizing their disordered eating as a stress management strategy (to both regulate negative affect and provide a sense of control) and acknowledged the multiple mechanisms through which stress can interfere with recovery. The results emphasise the importance of stress in formulations of eating disorders, as well as prioritizing the development of insight and adaptive coping mechanisms in interventions.

This abstract was presented in the **Learning from Consumers** stream of the 2014 ANZAED Conference.

